# Sex differences in neural correlates of common psychopathological symptoms in early adolescence

**DOI:** 10.1017/S0033291720005140

**Published:** 2022-10

**Authors:** Francesca Biondo, Charlotte Nymberg Thunell, Bing Xu, Congying Chu, Tianye Jia, Alex Ing, Erin Burke Quinlan, Nicole Tay, Tobias Banaschewski, Arun L. W. Bokde, Christian Büchel, Sylvane Desrivières, Herta Flor, Vincent Frouin, Hugh Garavan, Penny Gowland, Andreas Heinz, Bernd Ittermann, Jean-Luc Martinot, Hervé Lemaitre, Frauke Nees, Dimitri Papadopoulos Orfanos, Luise Poustka, Sabina Millenet, Juliane H. Fröhner, Michael N. Smolka, Henrik Walter, Robert Whelan, Edward D. Barker, Gunter Schumann, IMAGEN Consortium

**Affiliations:** 1 Department of Neuroimaging, Institute of Psychiatry, Psychology & Neuroscience, King's College London, UK; 2 Centre for Population Neuroscience and Stratified Medicine (PONS) and Social, Genetic and Developmental Psychiatry Centre, Institute of Psychiatry, Psychology & Neuroscience, King's College London, UK; 3 Swedish National Board of Health and Welfare, Socialstyrelsen, Stockholm, Sweden; 4 Institute of Science and Technology for Brain-Inspired Intelligence, Fudan University, Shanghai, China; 5 Key Laboratory of Computational Neuroscience and Brain-inspired Intelligence, Fudan University, Ministry of Education, China; 6 Department of Child and Adolescent Psychiatry and Psychotherapy, Central Institute of Mental Health, Medical Faculty Mannheim, Heidelberg University, Mannheim, Germany; 7 Discipline of Psychiatry, School of Medicine and Trinity College Institute of Neuroscience, Trinity College Dublin, Dublin, Ireland; 8 University Medical Centre Hamburg-Eppendorf, Germany; 9 Department of Cognitive and Clinical Neuroscience, Central Institute of Mental Health, Medical Faculty Mannheim, Heidelberg University, Mannheim, Germany; 10 Department of Psychology, School of Social Sciences, University of Mannheim, Germany; 11 NeuroSpin, CEA, Université Paris-Saclay, Gif-sur-Yvette, France; 12 Departments of Psychiatry and Psychology, University of Vermont, USA; 13 Sir Peter Mansfield Imaging Centre School of Physics and Astronomy, University of Nottingham, UK; 14 Department of Psychiatry and Psychotherapy, Campus Charité Mitte, Charité, Universitätsmedizin Berlin, Germany; 15 Physikalisch-Technische Bundesanstalt, Braunschweig and Berlin, Germany; 16 Institut National de la Santé et de la Recherche Médicale, INSERM U A10 ‘Trajectoires développementales en psychiatrie’, Université Paris-Saclay, Ecole Normale supérieure Paris-Saclay, CNRS, Centre Borelli, Gif sur Yvette, France; 17 Groupe d'Imagerie Neurofonctionnelle, Institut des Maladies Neurodégénératives, CNRS UMR 5293, Université de Bordeaux, Centre Broca Nouvelle-Aquitaine, Bordeaux, France; 18 Institute of Medical Psychology and Medical Sociology, University Medical Center Schleswig Holstein, Kiel University, Kiel, Germany; 19 Department of Child and Adolescent Psychiatry and Psychotherapy, University Medical Centre Göttingen, Germany; 20 Department of Psychiatry and Neuroimaging Center, Technische Universität Dresden, Germany; 21 School of Psychology and Global Brain Health Institute, Trinity College Dublin, Ireland; 22 Department of Psychology, Institute of Psychiatry, Psychology & Neuroscience, King's College London, UK; 23 PONS Research Group, Department of Psychiatry and Psychotherapy, Campus Charite Mitte, Humboldt University, Berlin and Leibniz Institute for Neurobiology, Magdeburg, Germany, and Institute for Science and Technology of Brain-inspired Intelligence (ISTBI), Fudan University, Shanghai, P.R. China

**Keywords:** Adolescence, attention-deficit hyperactivity disorder, externalizing symptoms, grey matter, IMAGEN, internalizing symptoms, psychopathology, sex differences, voxel-wise morphometry

## Abstract

**Background:**

Sex-related differences in psychopathology are known phenomena, with externalizing and internalizing symptoms typically more common in boys and girls, respectively. However, the neural correlates of these sex-by-psychopathology interactions are underinvestigated, particularly in adolescence.

**Methods:**

Participants were 14 years of age and part of the IMAGEN study, a large (*N* = 1526) community-based sample. To test for sex-by-psychopathology interactions in structural grey matter volume (GMV), we used whole-brain, voxel-wise neuroimaging analyses based on robust non-parametric methods. Psychopathological symptom data were derived from the Strengths and Difficulties Questionnaire (SDQ).

**Results:**

We found a sex-by-hyperactivity/inattention interaction in four brain clusters: right temporoparietal-opercular region (*p* < 0.01, Cohen's *d* = −0.24), bilateral anterior and mid-cingulum (*p* < 0.05, Cohen's *d* = −0.18), right cerebellum and fusiform (*p* < 0.05, Cohen's *d* = −0.20) and left frontal superior and middle gyri (*p* < 0.05, Cohen's *d* = −0.26). Higher symptoms of hyperactivity/inattention were associated with lower GMV in all four brain clusters in boys, and with higher GMV in the temporoparietal-opercular and cerebellar-fusiform clusters in girls.

**Conclusions:**

Using a large, sex-balanced and community-based sample, our study lends support to the idea that externalizing symptoms of hyperactivity/inattention may be associated with different neural structures in male and female adolescents. The brain regions we report have been associated with a myriad of important cognitive functions, in particular, attention, cognitive and motor control, and timing, that are potentially relevant to understand the behavioural manifestations of hyperactive and inattentive symptoms. This study highlights the importance of considering sex in our efforts to uncover mechanisms underlying psychopathology during adolescence.

Heterogeneity in mental disorders is a significant and largely unresolved problem that stands in the way of the development of predictors and targeted therapies. A clear example of this heterogeneity is sex-related, which is a ubiquitous phenomenon in psychopathology – many disorders show uneven sex distributions of prevalence, symptoms, age of onset and treatment response (Martel, 2013; Paus, Wong, Syme, & Pausova, 2017). For example, externalizing disorders are more frequently observed in males than females, whilst the reverse pattern is true for internalizing disorders (Carragher et al., 2016; Martel, 2013). Previous research suggests that brain differences in males and females may be related to differences in the expression of both externalizing and internalizing forms of psychopathology (Giedd, Castellanos, Rajapakse, Vaituzis, & Rapoport, 1997; Kaczkurkin, Raznahan, & Satterthwaite, 2019; Lenroot et al., 2007). This underscores the importance of identifying sex-by-psychopathology interaction patterns as manifested in the brain. The timing of the development of sex differences is important. Brain and behavioural sex differences are particularly noticeable during developmental stages such as childhood and adolescence (Paus et al., 2017). Moreover, although psychopathology can be observed early in childhood (Egger & Angold, 2006), widespread vulnerability to psychopathology becomes most apparent in adolescence (Paus, Keshavan, & Giedd, 2008; Schumann et al., 2010), with half of the lifetime psychopathological burden detectable by the mid-teens and 75% by the mid-20s (Gore et al., 2011; Kessler et al., 2007). Hence, adolescence is a time when both sex differences and psychopathology are marked, making adolescence an important window to investigate potential sex differences in the neural correlates of these disorders. Advancing the understanding of sex-bias in common psychopathology may aid in the development of effective screening, intervention strategies and health policy decisions.

Previous research attempts to uncover sex-by-psychopathology interactions in the brain exist. For example, depression and low positive attributes have been found to associate with lower grey matter (GM) in limbic regions in females (Kong et al., 2013) and lower GM in striatal regions in males (Kong et al., 2013). In conduct disorder, sex differences were reported in parietal and frontal regions with boys showing smaller cortical thickness, higher gyrification and higher surface area compared to controls, whilst girls showed the opposite pattern (Frere et al., 2020; Smaragdi et al., 2017). However, these previous studies are sparse and have various limitations that leave the search for sex-by-psychopathology interactions in the brain elusive. Limitations include power-related issues driven by small sample sizes and age or sex-dependent confounds driven by wide age ranges and unbalanced sex groups (Abi-Dargham & Horga, 2016; Kaczkurkin et al., 2019; Klein et al., 2017; Kong et al., 2013; Lenroot et al., 2007; Rutter, Caspi, & Moffitt, 2003; Smaragdi et al., 2017; Valera et al., 2010; Wu et al., 2019). Also, one of the dominant methodological approaches is region-of-interest (ROI) analyses, which whilst being informative, is less useful in generating new findings and hypotheses outside of the pre-selected ROIs. Voxel-based morphometry offers an alternative whole-brain approach that does not require the *a priori* selection of ROIs (Ashburner & Friston, 2000). Furthermore, these studies often rely on case–control designs, which assume a categorical distribution of psychopathology, an assumption that has been contested with data suggesting that symptoms may be spread over a spectrum instead (Kaczkurkin et al., 2019; Klein et al., 2017; Marquand, Rezek, Buitelaar, & Beckmann, 2016).

This study aims to investigate sex-by-psychopathology interactions in the brain structure of young adolescents. To do so we investigate GMV changes given that this brain measure has been previously associated with a myriad of psychiatric diagnoses (Goodkind et al., 2015). As a measure of psychopathology, we look at common externalizing and internalizing symptoms as these have been previously reported to show sex differences (Carragher et al., 2016; Martel, 2013). Importantly, this study overcomes the limitations of previous reports mentioned above by way of the sample characteristics and methodological approaches it employs. We utilise IMAGEN, a sex-balanced sample of approximately 2000 adolescents within a focused age range of around 14 years (Schumann et al., 2010). IMAGEN is a community-based sample that allows us to examine the symptoms of psychopathology as traits, which are assumed to be continuously, rather than categorically, distributed and hence are a closer representation of the distribution of symptoms in the general population (Kaczkurkin et al., 2019; Marquand et al., 2016). Considering the time between the occurrence of first symptoms and the first contact with psychiatric services generally spans several years, investigating symptoms rather than fully-fledged clinical cases may offer important novel insights into their aetiology. Importantly, instead of testing hypotheses via an ROI approach, we use an exploratory whole-brain method to maximise the discoverability of associated brain areas. At the same time, we use robust and conservative methods to minimise false positives, for example, by using non-parametric statistical brain analyses, correcting for multiple comparisons and including various covariates. Finally, a secondary aim of this study is to confirm whether sex differences exist at the symptom-behavioural level and we hypothesise that externalizing and internalizing symptoms will be higher in boys and girls, respectively.

## Methods

### Participants

**Fig. 1.**
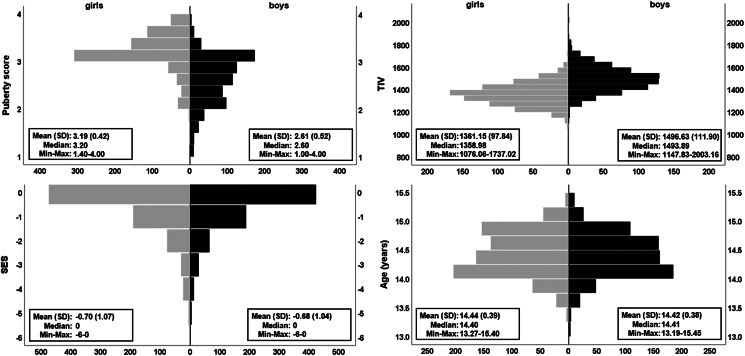
Distribution of covariates. The pyramid plots show the distribution of puberty score, TIV, SES and age split by sex (girls, *N* = 801 in grey; boys, *N* = 725 in black). Means, standard deviation (s.d.), median, observed minimum and maximum are presented for each raw (non-residualised) covariate. Handedness (not illustrated) was 89.4% right and 10.6% left/ambidextrous for girls and, 85.8% right and 14.2% left/ambidextrous for boys. Additional plots using the maximum available cases for behavioural data (*N* = 2046) can be found in online Supplementary Fig. S1.

The IMAGEN sample was recruited and tested across eight European sites. Recruitment was achieved via high schools with two broad aims: (i) reduce genetic diversity by maximizing European descent homogeneity, and (ii) maximise socioeconomic status (SES), emotional and cognitive development diversity by sampling equally from private, state-funded and special units. Details on recruitment and assessment can be found elsewhere (Schumann et al., 2010). At each site, the study was approved by the appropriate Ethics boards. Informed assent and consent were obtained from the participant and their legal guardian, respectively. The community-based sample comprised of adolescents aged 13.3–15.5 years (Fig. 1). Participants were removed if they had missing data for the following variables: psychopathological symptoms, puberty score, age, SES, if they displayed a verbal or reasoning IQ score below 75 on the WISC-IV or if neuroimaging data were either missing or failed quality control, reducing the original sample of *N* = 2090 to *N* = 1526 (Supplementary material). For the behavioural analyses alone, a larger subset of the data (*N* = 2046) was available (Supplementary material). The socioeconomic and housing subscale of the Family Stresses questionnaire (Goodman, Ford, Richards, Gatward, & Meltzer, 2000) was used as an index of SES (Supplementary material). Sex was self-reported (male or female). A puberty score was obtained from the Puberty Development Scale that is based on the development of external primary and secondary sex characteristics (Petersen, Crockett, Richards, & Boxer, 1988).

### Psychopathology

**Fig. 2.**
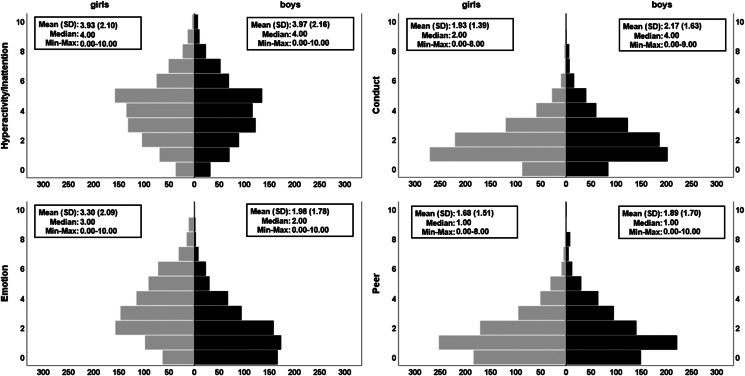
Distributions of the four psychopathological symptoms subscales of the SDQ. Pyramid plots are split by sex (girls, *N* = 801 in grey; boys, *N* = 725 in black). Significant sex differences were found for conduct problems (higher in boys), emotional difficulties (higher in girls) and peer problems (higher in boys). The plots show raw (non-residualised) versions of the variables. Full GLM details can be found in online Supplementary Table S3a. Additional variants of these plots and analyses can be found in Supplementary materials (online Supplementary Fig. S2, online Supplementary Tables S3b–S3c).

Psychopathological symptoms were assessed using the self-rated Strengths and Difficulties Questionnaire (SDQ) (Goodman, 1997), a brief behavioural screening tool with five subscales and five items per subscale (Supplementary material). We focused on the four subscales measuring difficult behaviour: hyperactivity/inattention, conduct problems, emotional problems and peer problems (Fig. 2). These can be split into two categories, externalizing symptoms (hyperactivity/inattention; conduct) and internalizing symptoms (emotional; peer) (Goodman, Lamping, & Ploubidis, 2010).

### Neuroimaging

The MRI scanners used across the eight sites were 3-Tesla and of various manufacturers (Siemens, Philips, GE, Bruker). To ensure comparability across sites, scan parameters were standardised to the highest degree possible and were optimised based on the ADNI1 protocol (http://adni.loni.usc.edu/methods/documents/mri-protocols/) to give good agreement in the final images despite scanner differences. T1-weighted images were spatially normalised and segmented into GM using a voxel-wise-based morphometry (VBM) pipeline involving unified segmentation (Ashburner & Friston, 2005), a diffeomorphic registration (Ashburner, 2007) within the SPM software (https://www.fil.ion.ucl.ac.uk/spm/) and smoothing to 8 mm (see Supplementary materials, for neuroimaging data acquisition and preprocessing details).

### Statistical analyses

To address the main research question of whether sex-by-psychopathology interactions are detected for GM outcomes, we ran four general linear models (GLM), one for each of the four psychopathological symptom subscale scores (hyperactivity/inattention, conduct, emotional and peer problems) with GMV at the voxel-level as the dependent variable. The sex-by-psychopathology interaction term was calculated by multiplying sex to the relevant subscale score and used as the covariate of interest. Sex, the relevant subscale score, site, handedness, age, puberty, SES and total intracranial volume (TIV) were added as covariates (Figs 1 and 2). Also, considering that the four psychopathological symptom subscales were significantly correlated with each other (online Supplementary Table S1), each one of the four GLMs included the remaining three subscales as covariates (design matrix: online Supplementary Fig. S3). A version of the analyses excluding the other subscales as covariates can be found in Supplementary materials. These GLMs were implemented across the whole brain, using voxel-wise analyses using a threshold-free cluster enhancement (TFCE) approach (Smith & Nichols, 2009). Cluster-wise inference is known to have higher sensitivity compared to voxel-wise tests when the signal is spatially distributed (Woo, Krishnan, & Wager, 2014). One common way to run cluster-wise analysis is via the SPM random-field approach, however, to avoid problems related to this method (Eklund, Nichols, & Knutsson, 2016) and avoid the arbitrariness of setting a cluster-forming threshold as well as ensure appropriate correction for the family-wise error (FWE), a robust non-parametric TFCE approach was used and implemented via FSL software's (https://fsl.fmrib.ox.ac.uk/fsl/fslwiki/) randomise function (Winkler, Ridgway, Webster, Smith, & Nichols, 2014). The null distribution was generated using 10 000 permutations of the experimental labelling with adjustment for the FWE using a threshold of *p* < 0.05. Given that our analysis was exploratory, we tested for both directed contrasts (negative and positive associations with GMV) and hence used two-tailed statistical significance values. In addition, we corrected for multiple comparisons experiment-wise (across the four behavioural symptom subscales) using Bonferroni correction by multiplying the *p* value by four (i.e. number of tests conducted).

To test the direction of any resulting interaction, a post-hoc test was run by splitting the sample by sex and running a GLM for each significant cluster, with GMV in each cluster as the dependent variable, the relevant psychopathology symptom subscale (hyperactivity/inattention) as the covariate of interest and site, handedness, age, puberty, SES, TIV and the remaining three psychopathology symptom subscales as covariates. To test if an interaction effect was also global, not just regional, a second post-hoc test used an analogous GLM on the whole sample using whole-brain GMV as the dependent variable and sex-by-hyperactivity/inattention as the covariate of interest. A third post-hoc test investigated whether the sex-by-hyperactivity/inattention GMV interaction within the significant clusters remained significant even when covarying for an additional measure of brain volume, normalised brain volume, corresponding to the sum of the volumes of grey and white matter, divided by TIV. See Supplementary materials for post-hoc test details.

Finally, a secondary analysis was carried out to investigate sex differences within each of the four subscale symptoms scores and test if, as expected from the literature, the externalizing symptoms subscale scores (hyperactivity/inattention, conduct) and internalizing symptoms subscale scores (emotional, peer problems) were higher in boys and girls, respectively. This was achieved using four, Bonferroni-corrected, GLMs with the relevant subscale score as the dependent variable, sex as the covariate of interest and site, handedness, age, puberty, SES, TIV and the remaining three subscale scores as covariates. GLMs that yielded null findings were supplemented with Bayesian linear regressions to quantify the probability of the null finding. A Bayesian framework is different from a frequentist (*p* value-oriented) approach. The former uses observed data to calculate probability estimates for different hypotheses, reflecting which of these has higher or lower credibility (Van Den Bergh et al., 2020). Bayes factors (BF) were calculated using the ‘lmBF’ function with default settings from the BayesFactor package (Morey & Rouder, 2018) in *R* (R Core Team, 2019). The Bayes quotient, which allows to make an inference on the relative importance of a covariate, was calculated by dividing the BF of a full model (all covariates) by the BF of a reduced model (all covariates minus sex).

## Results

**Fig. 3.**
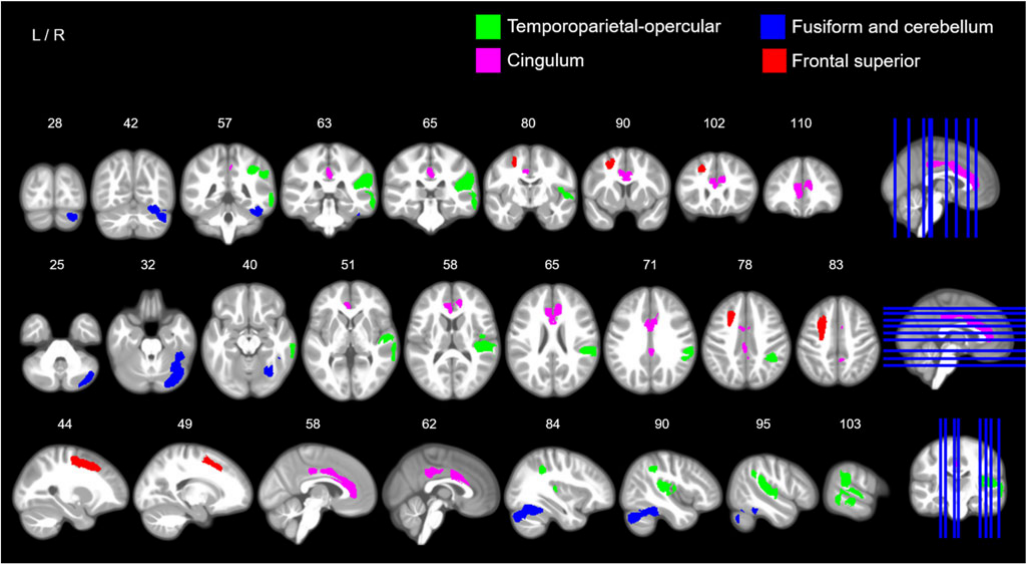
Sex-by-hyperactivity/inattention interaction in GMV. The image illustrates coronal (top row), axial (middle row) and sagittal slices (bottom row) with coloured maps representing binary maps for the four statistically significant clusters indicating a negative association between GMV and symptom scores of hyperactivity/inattention in boys, and a positive association in girls for the temporoparietal-opercular and fusiform and cerebellar clusters. Green = cluster 1, right temporoparietal-opercular region; pink = cluster 2, cingulum; blue = cluster 3, right fusiform and cerebellum; red = cluster 4, frontal superior. For detailed anatomical descriptions, refer to Table 2 and the ‘Results’ section. For an unthresholded map and non-significant clusters, see Supplementary materials (online Supplementary Figs S5–S6). Maps are overlaid on a T1-weighted brain image made from 500 T1-weighted scans from the study sample. Images were produced using MRIcron (www.nitrc.org/projects/mricron).

We report a sex-by-psychopathology interaction in GMV with hyperactivity/inattention problems in four brain clusters: (i) a right cluster including the superior temporal gyrus and extending to the supramarginal, inferior parietal Heschl gyrus, and rolandic operculum; (ii) a frontal medial cluster including the bilateral cingulum anterior and extending to the mid-cingulum; (iii) a right cluster involving the cerebellum, fusiform and the inferior temporal gyrus; (iv) a left frontal cluster including the superior and middle frontal gyri and extending to the precentral gyrus (Table 1, Fig. 3, see online Supplementary Fig. S5 for the unthresholded statistical map). We report two additional small, left clusters; however, these did not survive correction for multiple comparisons (experiment-wise) (Table 1). Excluding the other psychopathology symptom subscales as covariates in the analysis did not change the overall pattern of our findings (online Supplementary Tables S2, S3c, online Supplementary Fig. S4). The GLMs using the remaining three psychopathological symptoms subscales (conduct, emotion and peer problems) did not show any significant sex-by-psychopathology interactions in GMV.

**Table 1.** Sex-by-hyperactivity/inattention interaction in voxel-wise GMV

Cluster #		Right/left	BA	Peak *t* values' coordinates			Peak *t* values	*k*	Unadjusted *p* value	Adjusted *p* value	Effect size (Cohen's *d*)
1	Temporal superior	R	42	57	−33	21	−4.68	6035	0.002	0.008**	−0.24
2	Cingulum anterior	L	24	−5	33	11	−3.5	4487	0.010	0.040*	−0.18
3	Cerebellum crus I	R	19	42	−78	−33	−3.9	4266	0.006	0.024*	−0.20
4	Frontal superior	L	32	−18	20	42	−4.97	1771	0.003	0.012*	−0.26
5	Frontal superior	L	32	−21	36	26	−4.02	74	0.022	0.088	−0.20
6	Frontal middle	L	46	−23	45	11	−3.91	27	0.024	0.096	−0.20

**Fig. 4.**
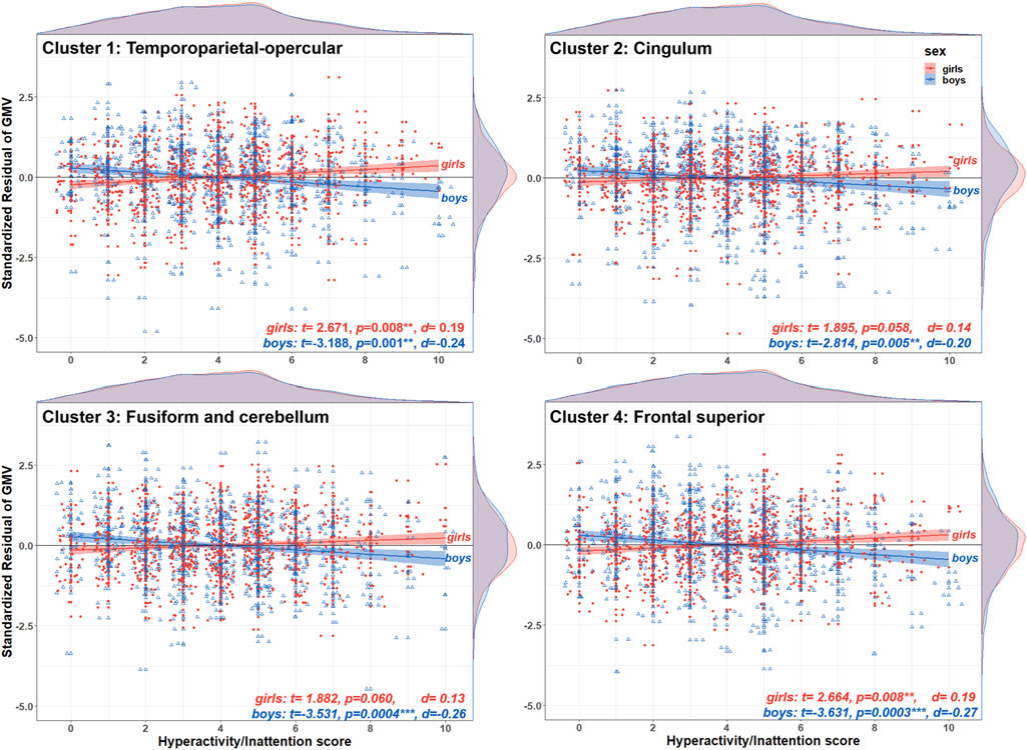
Direction of the sex-by-hyperactivity/inattention interaction in voxel-wise GMV. The scatterplots illustrate the residual of GMV on the *y*-axis for each one of the four brain clusters showing a significant sex-by-hyperactivity/inattention interaction, and hyperactivity/inattention symptoms score on the *x*-axis, stratified by sex (girls in filled red circles; boys in empty blue triangles). At the top and right side of each scatterplot are density plots illustrating the distribution of hyperactivity/inattention symptoms score and standardised residuals, respectively. For boys, there was a negative association between hyperactivity/inattention symptoms and GMV in all four brain regions (negative *t*-values, *p* < 0.05). For girls, this association was positive but statistically significant for only the temporoparietal-opercular and frontal superior clusters (positive *t* values, *p* < 0.05). The standardised residuals were calculated using a GLM with GMV at each cluster as the dependent variable and sex, site, handedness, age, puberty, SES, TIV, conduct, peer and emotion problems as covariates.

This table shows the six brain clusters that resulted from a voxel-wise analysis with a TFCE approach, indicating sex-by-hyperactivity interactions in GMV which were statistically significant for the first four clusters. In boys, a negative association between hyperactivity and inattentive symptoms and GMV was found in all four brain regions. In girls, the association was positive and significantly so in the temporal superior and cerebellar-fusiform clusters (see Fig. 4 for post-hoc tests revealing the direction of these interactions). Unadjusted *p* values are corrected for the FWE within the brain statistical map at an *α* = 0.05, adjusted *p* values are additionally corrected for experiment-wise multiple comparisons across the four subscales using Bonferroni correction. The adjusted *p* values that are statistically significant are marked with ** (*p* < 0.01) and * (*p* < 0.05). Anatomical descriptions were achieved using the AAL (Anatomical Automatic Labelling) and BA (Brodmann Area) atlases provided in MRIcron v.2016 (www.nitrc.org/projects/mricron) (Rorden, Karnath, & Bonilha, 2007). For additional details see the ‘Results’ section. *k* = cluster size; degrees of freedom (df) = 1507; *p* value (2-tailed) with *α* = 0.05.

Post-hoc tests indicated that the associations between hyperactivity/inattentive symptoms and GMV were negative for boys (negative *t*-statistics with a Cohen's *d* ranging from *d* = −0.20 to *d* = −0.27), positive for girls (positive *t*-statistics, *d* = 0.13 to *d* = 0.19) and the directions of these associations were significant for all clusters (*p* < 0.05) except for two clusters for girls, the fusiform-cerebellar and cingulum, which were at borderline significance (Fig. 4). The second post-hoc test revealed that sex-by-hyperactivity was significantly associated with whole-brain GMV (*β* = −0.86, *p* = 0.0025, *d* = −0.16). The third post-hoc test showed that sex-by-hyperactivity/inattention interaction in GMV within the significant clusters remained significant even when covarying for normalised brain volume (*β* = −6.22, *p* = 6.27 × 10^−10^, *d* = −0.32). The latter result suggests that the main findings at the voxel-level were sufficiently robust, surviving additional brain volume correction.

The secondary analysis, which tested sex differences in each psychopathological symptoms subscale, revealed that conduct problems (*β* = 0.13, *r* = 0.12, *d* = 0.24, *p*_adj_ = 1.20 × 10^−5^) and peer problems were significantly higher in boys (*β* = 0.17, *r* = 0.15, *d* = 0.30, *p*_adj_ = 2.21 × 10^−8^) compared to girls, whilst emotional problems were higher in girls (*β* = −0.36, *r* = −0.33, *d* = −0.69, *p*_adj_ = 6.15 × 10^−39^) compared to boys (Fig. 2). The hyperactivity/inattention problems subscale did not show a significant sex difference and using the maximum number of complete cases available for behavioural data and/or excluding psychopathology subscales as covariates did not change the overall pattern of our findings (online Supplementary Tables S3a–S3c). A Bayesian version of the latter analysis revealed that the data are 5.73 times more likely under the reduced model (no sex), compared to the full model. This suggests that sex is not a meaningful predictor of hyperactivity/inattention whilst accounting for the other covariates.

## Discussion

We investigated the neural correlates of sex differences of common psychopathological symptoms in 14-year-old adolescents. For the first time, using a whole-brain approach within a large-scale, sex-balanced, community-based sample, we found a sex-by-psychopathology interaction effect. This main finding indicated a negative association between GMV in four brain regions and hyperactivity/inattention symptoms in boys, and a positive association in two out of these four regions in girls. A secondary set of findings includes sex differences in symptoms of psychopathology, with higher scores for boys in conduct and peer problems and higher scores for girls in emotional problems. All results reflect associations with psychopathology symptoms subscales that have been covaried out of the other three subscales. Nonetheless, excluding these covariates in the analyses did not change the overall pattern of our findings. The differences in GMV that we report may reflect impaired processing in functions associated with the affected brain regions, including the manifestation of the observed hyperactive and inattentive symptoms. However, our study does not permit making any direct brain–behaviour links (Poldrack, 2011) and these GMV differences may be due to other reasons, for example, as a consequence rather than a cause of these symptoms. What follows are descriptions of the behavioural relevance of our brain results and given the sparsity of sex-by-hyperactivity/inattention findings, we interpret these with respect to the wider literature on attention-deficit hyperactivity disorder (ADHD).

The first brain cluster involved a right temporoparietal-opercular region. Abnormalities in this region (amongst others) have been previously reported in both structural neuroimaging studies on ADHD showing lower GMV (Kobel et al., 2010; Villemonteix et al., 2015*a*), as well as functional neuroimaging studies on ADHD showing decreased activation during attentional tasks (Hart, Radua, Nakao, Mataix-Cols, & Rubia, 2013). One important function associated with the temporoparietal-opercular region is attentional processing (Corbetta & Shulman, 2002). Also, other studies have previously linked abnormalities in this region to a reduced ability to ignore distractors and detect salient stimuli (Kobel et al., 2010; Rubia, Smith, Brammer, & Taylor, 2007) hence fitting with symptoms of inattention. Another function associated with this region is secondary sensorimotor processing, whereby sensory (e.g. tactile) stimuli are integrated with cognitive control (Chen et al., 2008), which are also disrupted in ADHD (McLeod, Langevin, Goodyear, & Dewey, 2014). Of note, this result corroborates findings from healthy brain development that indicate less GMV in this brain region (amongst others), in boys when compared to girls (Ruigrok et al., 2014). This may suggest the possibility of an ‘extreme male brain’ developmental trajectory playing a part in explaining our finding (Baron-Cohen et al., 2011) such that biological ‘maleness’ (e.g. exposure to foetal testosterone, expression of the Y chromosome) may lead to structural differences (e.g. via decreased GMV in the temporoparietal-opercular region), which in turn increases the risk of exhibiting certain, in this case, inattentive and hyperactive behaviours. However, our findings are insufficient to add weight to this hypothesis and further investigations are warranted.

The second cluster involved the anterior and mid-cingulum. Core abnormalities in the cingulum have been previously reported for individuals with ADHD (Bonath, Tegelbeckers, Wilke, Flechtner, & Krauel, 2018; Bralten et al., 2016; Bush et al., 1999; Dirlikov et al., 2015; Frodl & Skokauskas, 2012; Liddle et al., 2011; McLeod et al., 2014) including a sex-by-ADHD interaction in boys (Villemonteix et al., 2015*b*). Recent developments suggest that the cingulum is a key component in a distributed network in decision making and high-level control of action (Heilbronner & Hayden, 2016; Hunt & Hayden, 2017) including monitoring (e.g. error monitoring), controlling (e.g. response selection and motor control) and evaluating value. Hence, disruption of these high-level operations is consistent with the attentional and impulsive impairments of ADHD (Bush et al., 1999). We further note that together, the anterior cingulum and the right temporoparietal-opercular region, in particular the insula, overlap with the salience network, which is associated with the ability to detect salient stimuli and to task engagement (Menon & Uddin, 2010). This network has been previously found to be abnormal in participants with ADHD (Rubia et al., 2007, 2009).

The third cluster includes the right cerebellum and right fusiform gyrus. Abnormalities in these regions have been widely implicated in the impairments of motor and cognitive control, attention and timing in ADHD (Bonath et al., 2018; Castellanos & Proal, 2012; del Campo, Müller, & Sahakian, 2012; Giedd, Blumenthal, Molloy, & Castellanos, 2001; Hart et al., 2013; Noreika, Falter, & Rubia, 2013; Valera, Faraone, Murray, & Seidman, 2007). In particular, abnormal activities in the cerebellum and fusiform (Hart et al., 2013; Rubia et al., 2009; Valera et al., 2010) have been linked to compensatory attentional activity in ADHD (Hart et al., 2013; Rubia et al., 2009). One possibility is that lower GMV in visual processing areas, such as the fusiform, may reflect deficient modulations of the visual system, for example, by decreased top-down influence on perceptual processes (Sigi Hale et al., 2014; Yalachkov, Kaiser, & Naumer, 2010).

The fourth cluster involved the left middle and superior parts of the frontal gyrus extending to the precentral gyrus. This region is important for higher-order functions, the so-called executive functions, particularly in controlling behaviour in the context of conflicting stimuli, such as the Stroop task (Egner & Hirsch, 2005; Mansouri, Tanaka, & Buckley, 2009) but also for controlling more basic motor functions. In the ADHD literature, lower GM in frontal lobes, including lateral and premotor regions, has been previously reported (Castellanos & Proal, 2012; Dirlikov et al., 2015; Jarczok, Haase, Bluschke, Thiemann, & Bender, 2019; Kumar, Arya, & Agarwal, 2017; Mostofsky, Cooper, Kates, Denckla, & Kaufmann, 2002; Seidman et al., 2006). Both executive dysfunction and impairments in motor response inhibition are relevant to symptoms of inattention and hyperactivity and core deficits in ADHD (Castellanos & Proal, 2012; Rubia, Smith, & Taylor, 2007; Sergeant, Geurts, Huijbregts, Scheres, & Oosterlaan, 2003).

Our findings have both novel and common aspects of previous research. To the best of our knowledge, there are only three studies on sex-by-ADHD interactions in the brain that have used whole-brain approaches. The first study did not report any sex-by-ADHD interactions at all (Castellanos et al., 2002). The second study was small (*N* = 33) (Villemonteix et al., 2015b) and is closest to our findings, reporting a sex-by-ADHD interaction indicating lower GMV in the cingulum for boys (which we replicate) and also higher GMV in the same region in girls (which emerged as a trend in our results). The third study (Wu et al., 2019) was a multimodal analysis whereby a complex sex-by-ADHD interaction was reported showing, among other neuroimaging measures, higher GMV in frontal areas and lower GMV in posterior brain regions, for girls with ADHD compared to controls (girls), but the reverse for boys with ADHD compared to controls (boys). However, this study was limited given it only had nine female participants with ADHD. ROI approaches to sex-by-ADHD interactions are also sparse and show limited convergence with our results. For example, Dirlikov et al. (2015) report lower surface area in precentral regions for boys (like us, although we looked at volume, not surface area), but not for girls for whom they found lower surface area in the prefrontal cortex (whilst we found higher GMV in the left frontal gyrus). However, unlike some other studies, we do not find any subcortical abnormalities (Qiu et al., 2009; Seymour et al., 2017; Wang et al., 2018). Nor did we detect any significant negative sex-by-ADHD interactions for girls (Dirlikov et al., 2015; Mahone et al., 2011). Overall, the four brain clusters fall within a subset of brain regions that have been previously associated with ADHD studies, which did not test for sex differences possibly because most participants in these studies were male (Frodl & Skokauskas, 2012; Kaczkurkin et al., 2019; Kumar et al., 2017; Valera et al., 2010). The effect sizes we report are small (Cohen's *d* of −0.18 to −0.26) but comparable and nominally larger than mega-studies on structural brain differences in ADHD (Boedhoe et al., 2020; Hoogman et al., 2017).

Our secondary analyses revealed sex differences at the phenotypic-symptom level. Conduct problems (externalizing) were higher in boys and emotional difficulties (internalizing) higher in girls. These results are congruent with the existing literature indicating that externalizing disorders are more frequent in boys and internalizing disorders are more frequent in girls (Becker et al., 2018; Carragher et al., 2016; Martel, 2013; Rutter et al., 2003). However, the remaining two psychopathology subscale scores did not replicate in a similar way. We expected peer relationship problems (internalizing) to be either the same across sexes (Becker et al., 2018) or higher in girls (Goodman et al., 2010) but instead found they were higher in boys. Furthermore, we found no sex differences for hyperactivity/inattention symptoms (externalizing). One possibility is that these unexpected results may be due to specific study characteristics (e.g. sample type, measures used). Whilst null findings do not imply an absence of effect and should be interpreted with caution, a Bayesian version of this analysis supported this null finding and, other studies using similar study characteristics also failed to detect sex differences in behavioural hyperactivity/inattention symptoms (Becker et al., 2018; Lundh, Wangby-Lundh, & Bjärehed, 2008). Similar to these two studies, we used the self-rated version of the SDQ hyperactivity/inattention measure, which does not correlate highly with the parent-rated version (Du Rietz et al., 2017). In addition, our results show a normalised (rather than a right-skewed) distribution for hyperactivity/inattention symptoms. The Lundh et al. (2008) study, which like ours is also a community-based sample, reports almost identical statistics to ours (means and standard deviations) for the hyperactivity/inattention subscale (although we cannot comment on the shape of the distribution as it is not included in their report). Considering the unexpected direction of the peer problems result and failure to detect any difference in the hyperactivity/inattention symptoms similar to the Lundh et al. (2008) and Becker et al. (2018) studies, this may suggest that a sex-related externalizing–internalizing dichotomy may not be so clear-cut.

### Clinical implications

Clinically, our findings have two impacts. First, they inform the clinician that there are biological differences across the sexes in terms of hyperactivity/inattention symptoms. Second, they provide a lead to translational research. This includes research aimed at intervention practices, for example, pharmacological treatment as well as research on biomarkers of hyperactivity/inattention specific to boys and girls. A biomarker would aid the stratification of inattentive and hyperactive type of disorders by including a more objective biological-based approach instead of one only based on behavioural symptoms (Abi-Dargham & Horga, 2016). In addition, our findings are key to research aimed at uncovering mechanistic accounts of how psychopathological symptoms differ across sex and, in particular, as they do so along a continuum of psychopathology. Given that the assumptions for case–control designs are imperfect (e.g. well-defined grouping of cases *v.* controls, Marquand et al., 2016), investigating symptoms using a dimensional approach that spans from healthy to unhealthy is important as it is more representative of the manifestation of symptoms in the general population (Kaczkurkin et al., 2019).

### Limitations

Sex information was limited to biological sex and did not include any gender information, potentially limiting the interpretability of the results (Krieger, 2003). Also, our non-clinical sample, having sparser psychopathology than a clinical sample, may not show sex-by-psychopathology interactions that other studies report (Kong et al., 2013; Smaragdi et al., 2017). In turn, sparser psychopathology makes it reasonable to assume that the presence of current treatment is minimal, which is advantageous, as it minimises additional confounds. However, data on medication were unavailable, hence this study cannot exclude any treatment effects on observed results (Frodl & Skokauskas, 2012; Villemonteix et al., 2015*a*). Furthermore, the failure to detect other sex-by-internalizing symptoms interaction effects in GMV may be, in part, due to these appearing later in adolescence. Finally, the brain regions reported in this study are likely important for a large number of behavioural and cognitive functions that go beyond the ones discussed and that may vary in degree of relevance to hyperactivity and inattention.

## Conclusion

The brain regions we have identified via a sex-by-hyperactivity/inattention interaction effect are the first to be reported from a large, sex-balanced and community-based sample, using an exploratory whole-brain VBM approach. These study characteristics make our study not only powered to confirm previous findings but also to explore new ones and, within a sample that is more representative of the distribution of psychopathological symptoms in the general population. By addressing these common issues of previous studies, our results are valuable as they offer a reliable reference on sex-by-psychopathology interactions in GMV. Overall, these findings provide important leads for future research on mechanistic accounts of psychopathology and their sex-biases that are crucial to the advancement of improved targeted early interventions.

## Supplementary material

For supplementary material accompanying this paper visit https://doi.org/10.1017/S0033291720005140. click here to view supplementary material
