# Supplementary material for: Sex differences in neural correlates of common psychopathological symptoms in early adolescence
Source: Psychol Med. 2021 Mar 26;52(14):3086–96. doi: 10.1017/S0033291720005140 (PMC9693717; doi:10.1017/S0033291720005140)
Supplement: Supplementary file 1 [file S0033291720005140sup001.docx]

# Supplementary material

## Outline

[Part 1: Quality control, data preprocessing and questionnaires](#_Part_1:_Quality)

[Part 2: Distribution of independent variables with a larger subset of data (N=2046, not N=1526)](#_Part_2:_Distribution)

[Part 3: Design matrix for the main analysis](#_Part_3:_Design)

[Part 4: Additional results with variants of the analyses (larger subset (N=2046) and/or excluding other psychopathology symptoms subscales as covariates)](#_Part_4:_Additional)

[Part 5: Post-hoc tests](#_Part_5:_Post-hoc)

[References](#_References)

# Part 1: Quality control, data preprocessing and questionnaires

## Quality control (QC) and data filtering

Visual quality controls were run on the T1-weighted brain imaging data, to exclude scans that were compromised due to imaging artefacts. There were a total of 2090 T1 scans available, 433 of which failed QC and the remaining 1657 passed QC. Also, participants were removed if they had missing data for the following variables: Strengths and Difficulties Questionnaire (SDQ), puberty score, age, SES (via the Family Stresses Questionnaire) or if they displayed a verbal or reasoning IQ score below 75 on the WISC-IV(["WISC-IV," 2003](#_ENREF_7)). Out of the 1657 scans, 23 were removed because of missing puberty data, 63 were removed because of missing verbal IQ data, 17 were removed because verbal IQ was less than 75,16 were removed because reasoning IQ was less than 75,1 was removed because of missing age data, 3 were removed because of missing SDQ data and 8 were removed because of missing SES data, resulting in a total of N=1526 of complete cases for the main neuroimaging analysis. In the main paper we present behavioural analyses for this same sample size of N=1526, however in this supplementary document we also present results for analogous analyses for the maximum complete cases for behavioural data, N=2046 (**Part 2** & **Part 4** of this supplement). Analyses using the larger subset of the data did not alter the main findings of the paper.

## MRI data acquisition and preprocessing of GM data

The MRI scanners used across the 8 sites were 3-Tesla and of various manufacturers (Siemens, Philips, GE, Bruker). To ensure comparability across sites, scan parameters were standardized to the highest degree possible. Phantom scans and then in-vivo scans were then used to assess signal to noise and contrast to noise ratios at each site.

### T1 **Acquisition**

High resolution, T1-weighted images were obtained using a whole-brain 3D T1-weighted scan based on the ADNI1 protocol (<http://adni.loni.usc.edu/methods/documents/mri-protocols/>). The parameters were not identical across sites and were optimized by ADNI1 to give good agreement in the final images despite scanner differences (sagittal slice plane; repetition time: 2.3 s; echo time 2.8 ms; flip angle 8°; 256×256×160 matrix; isotropic voxel size: 1.1 mm).

### T1 preprocessing (Voxel-Based Morphometry (VBM) analysis)

T1-weighted scans were preprocessed using the 2008 version of the VBM toolbox (VBM8) (<http://dbm.neuro.uni-jena.de/vbm/>) running in SPM8 (v.5236)([Penny, Friston, Ashburner, Kiebel, & Nichols, 2007](#_ENREF_5)). T1-weighted images were spatially normalized into MNI space and segmented into GM and white matter (WM) maps using unified segmentation ([Ashburner & Friston, 2005](#_ENREF_2)) and diffeomorphic registration (Dartel) ([Ashburner, 2007](#_ENREF_1)) within the SPM software (<https://www.fil.ion.ucl.ac.uk/spm/>). One advantage of Dartel is that it creates study-specific templates which are particularly relevant to our investigation given the demographic differences of our adolescent population from standard adult templates. The modulated and warped GM segmentations were then smoothed using an isotropic 8 mm full width at half maximum Gaussian smoothing kernel. These GM maps were then used in all further statistical analyses. Using the VBM8 toolbox, global GM volume, global WM volume and global cerebrospinal fluid (CSF) were extracted for each participant. Total Intracranial Volume (TIV) for each participant was calculated as the sum of their GM, WM and CFS volumes. TIV was used as a covariate in the brain analyses to control for brain size.

## SES via the Family Stresses questionnaire

A subsection of the Family Stresses questionnaire was used as an index of socioeconomic status (SES). The Family Stresses questionnaire is part of the Development and Well-Being Assessment ([Goodman, Ford, Richards, Gatward, & Meltzer, 2000](#_ENREF_4)). The Family Stresses questionnaire has 16 items (see below) and the participant’s parents or guardian are asked to mark each item as either ‘No, or doesn’t apply’, ‘A little’ or ‘A lot’ which correspond to 0,1,2 points, respectively. There are four subscales: health score, relationship/addiction score, socioeconomic/housing score and work/pressure score. The socioeconomic/housing score, which is the sum of items 1,3,4,5 (***in italics and bold***, below) was used as the SES for each participant. To provide a more intuitive scale for SES the score was multiplied by -1 so that high scores (max 0) reflected high SES whilst low scores (max 8) reflected low SES.

1. ***You or your partner are unemployed***
2. You or your partner's work situation (pressure, hours, relationship with boss or colleagues)
3. ***Financial difficulties***
4. ***Home inadequate for the family's needs***
5. ***Problems with neighbours or the neighbourhood***
6. Too much to do (time pressures)
7. Lack of support from family and friends
8. Quarrels between the children in the family
9. Rows between the children and adults in the family
10. Tension between you and your partner
11. Tension between you and your ex-partner
12. Problems with your or your partner's physical health
13. Problems with your or your partner's psychological health
14. Other people being seriously ill, e.g. your parents
15. Alcohol or drug use by a family member
16. Gambling by a family member

No difference was found for SES across sex, Median girls = 0, Median boys = 0 (see **Figure 1**), Mann-Whitney *U*=290,771, *p* = .96. However, Spearman correlations revealed statistically significant associations of small effect size between SES and global GMV (ρ=.10, *p*=.00014) as well as associations between SES and the majority of the psychopathology symptom scores (hyperactivity/inattention: *ρ*=-.10, *p*=.00014, conduct: *ρ*=-.09, *p*=.00063; peer problems: *ρ*=-.06, *p*=.02 but not in emotion difficulties: *ρ*=-.04, *p*=.15 n.s.) Given the significant relationship between the SES and both the dependent variable and the covariate of interest, SES was added as a covariate to all analyses.

## Strengths and Difficulties Questionnaire (SDQ)

The SDQ([Goodman, 1997](#_ENREF_3)) can be downloaded from [www.sdqinfo.com](http://www.sdqinfo.com). The items from the four problem behaviour subscales (prosocial subscale was excluded from our study) are listed below ordered by subscale (see website for questionnaire order). Considering our focus on psychopathology, we ignored the fifth subscale of pro-social behaviour. For each item, the participant marks whether it’s “Not True, Somewhat True and Certainly True”. Details on scoring can be found on the website.

### Hyperactivity/inattention scale

1. I am restless, I cannot stay still for long
2. I am constantly fidgeting or squirming
3. I am easily distracted, I find it difficult to concentrate
4. I think before I do things
5. I finish the work I'm doing. My attention is good

### Conduct problems scale

1. I get very angry and often lose my temper
2. I usually do as I am told
3. I fight a lot. I can make other people do what I want
4. I am often accused of lying or cheating
5. I take things that are not mine from home, school or elsewhere

### Emotional problems scale

1. I get a lot of headaches, stomach-aches or sickness
2. I worry a lot
3. I am often unhappy, down-hearted or tearful
4. I am nervous in new situations. I easily lose confidence
5. I have many fears, I am easily scared

### Peer problems scale

1. I am usually on my own. I generally play alone or keep to myself
2. I have one good friend or more
3. Other people my age generally like me
4. Other children or young people pick on me or bully me
5. I get on better with adults than with people my own age

# Part 2: Distribution of independent variables with a larger subset of data (N=2046, not N=1526)


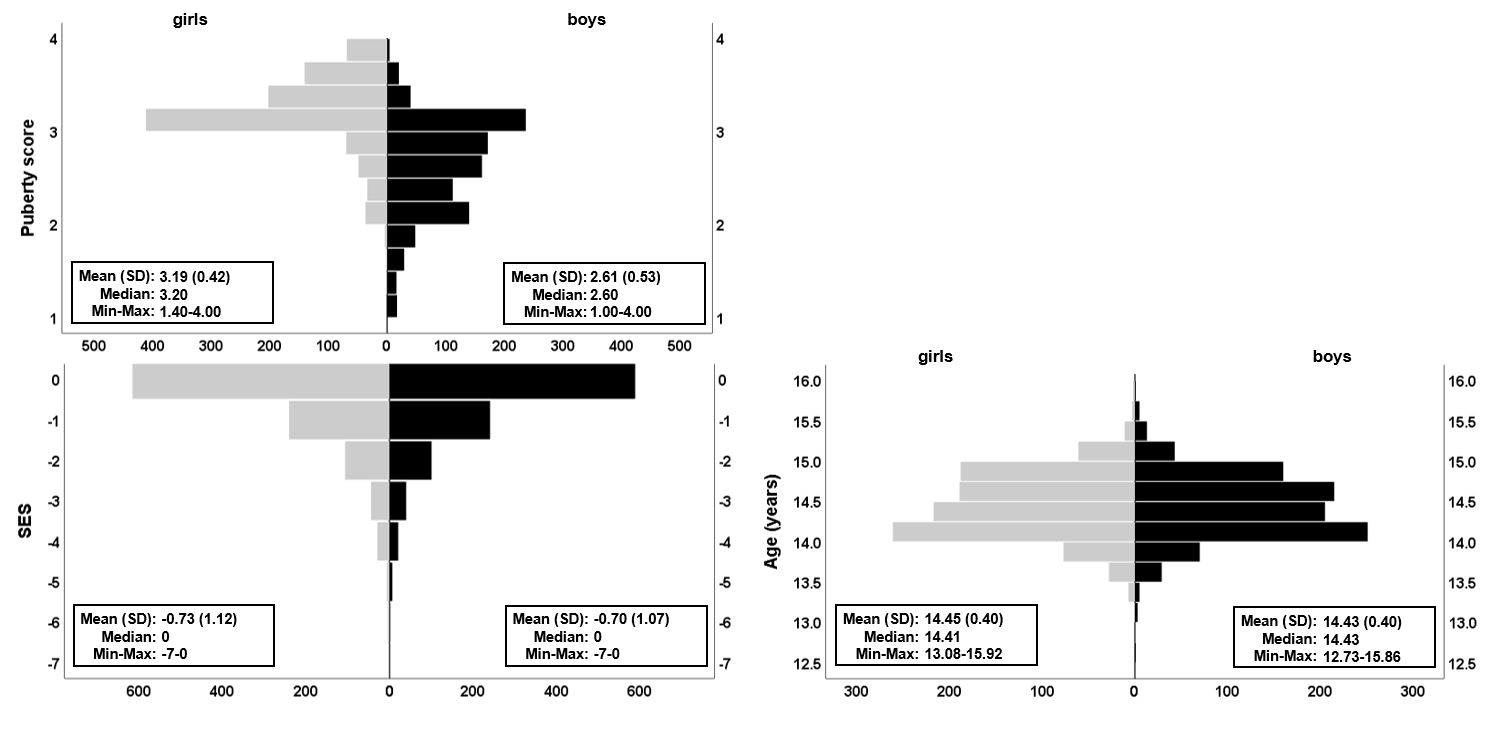


Figure S1 Distribution of covariates using pyramid plots. This figure is analogous to Figure 1 but using the maximum number of complete cases available for behavioural data, which gives a total of N=2046 (split by sex: girls, N=1045, in grey; boys, N=1001, in black). Means, standard deviation (SD), median, observed minimum and maximum are embedded in the plots for each raw (non-residualised) covariate. Handedness (not illustrated) was 89.3% Right and 10.7% Left/Ambidextrous for girls and 86.2% Right and 13.8% Left/Ambidextrous for boys.


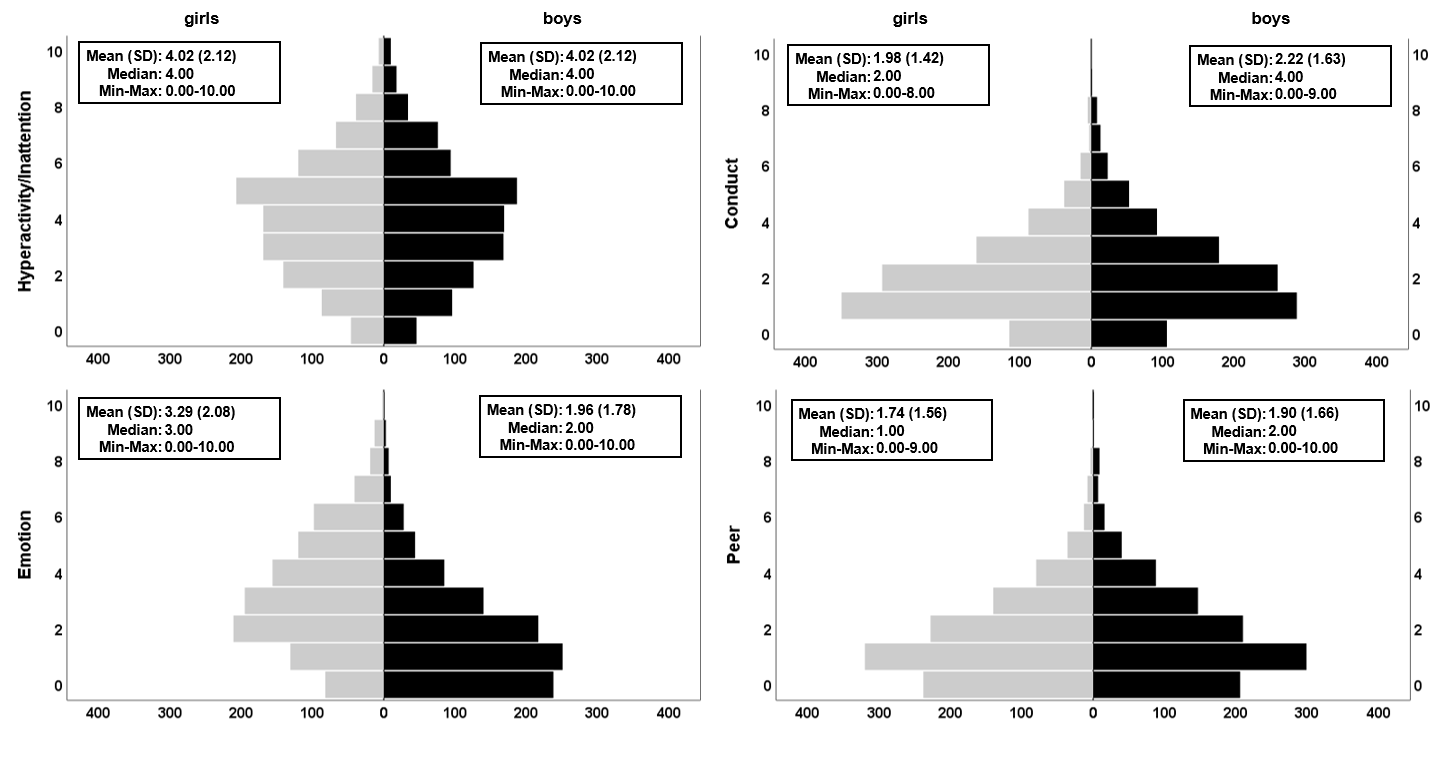


**Figure S2** **Distributions of the four psychopathological symptom subscales of the SDQ, split by sex.** This figure is analogous to **Figure 2** but using the maximum number of complete cases available for behavioural data, which gives a total of N=2046 cases (split by sex: girls, N=1045, in grey; boys, N=1001, in black). The variables shown are in the raw (non-residualised) format. Like the smaller subset of the data presented in the main paper (N=1526), significant sex differences were found for Conduct problems (higher in boys), Emotional difficulties (higher in girls) and Peer problems (higher in boys). Full GLM details can be found in **Table S2b**.

# Part 3: Design matrix for the main analysis

For the sex-by-psychopathology interactions in GMV analyses, the interaction term between sex and subscale score was calculated by multiplying sex to the relevant subscale score. In addition, sex and the relevant subscale score were added as covariates. For example, see **Figure S1** below.


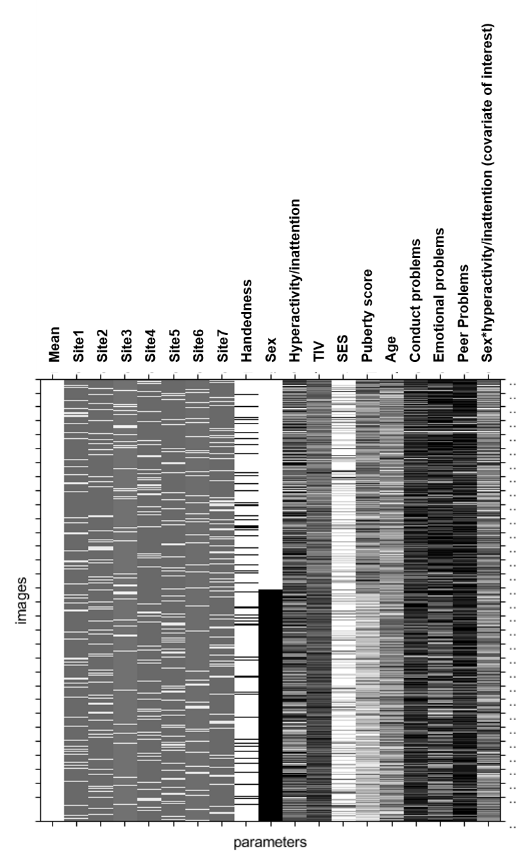


**Figure S3 The design matrix for the sex-by-hyperactivity/inattention TFCE analysis.** The dependent variables were the voxel-wise GMVs, the covariate of interest was sex-by-hyperactivity/inattention and the other covariates were the following: site variables (seven in total), handedness, sex, hyperactivity/inattention, TIV, SES, puberty score, conduct problems, emotional problems and peer problems.

# Part 4: Additional results with variants of the analyses

(Variants: larger subset (N=2046) and/or excluding other psychopathology symptoms subscales as covariates).

Table S1 Correlation matrix of the four psychopathological symptoms subscales of the SDQ.

|  |  | **N=1526** | | | | **N=2046** | | | | |
| --- | --- | --- | --- | --- | --- | --- | --- | --- | --- | --- |
|  | | **Hyperactivity/ Inattention** | **Conduct** | **Emotion** | **Peer** | | **Hyperactivity/ Inattention** | **Conduct** | **Emotion** | **Peer** |
| **Hyperactivity/ Inattention** | ***ρ*** |  | .365^***^ | .147^***^ | 0.035 | |  | .374^***^ | .172^***^ | .051^*^ |
|  | ***p*-value** |  | 3.42E-49 | 7.09E-09 | 1.74E-01 | |  | 7.54E-69 | 4.01E-15 | 2.07E-02 |
| **Conduct** | ***ρ*** |  |  | .148^***^ | .107^***^ | |  |  | .160^***^ | .133^***^ |
|  | ***p*-value** |  |  | 6.49E-09 | 2.79E-05 | |  |  | 3.32E-13 | 1.41E-09 |
| **Emotion** | ***ρ*** |  |  |  | .228^***^ | |  |  |  | .226^***^ |
|  | ***p*-value** |  |  |  | 2.21E-19 | |  |  |  | 3.97E-25 |

This table indicates that all subscales were significantly intercorrelated except for Hyperactivity/Inattention and Peer problems, which were only significantly correlated to each other when using the larger subset of N=2046. ***ρ* = Spearman’s rho; *p*-values are 2-tailed, ^***^ *p*-value<0.001, ^*^*p*-value<0.05**

**Table S2 Sex-by-hyperactivity/inattention interaction in GMV excluding other psychopathology symptom subscales as covariates.**

|  |  |  | **BA** | **Peak T values' coordinates** | | | **Peak T values** | **k** | ***unadjusted p*-value** | ***adjusted p-value*** |  |
| --- | --- | --- | --- | --- | --- | --- | --- | --- | --- | --- | --- |
| **Cluster #** |  | **Right/Left** |  |  |  |  |  |  |  |  | ***effect size (Cohen’s d)*** |
| 1 | Temporal Sup. | R | 42 | 55.5 | -33 | 19.5 | 4.74 | 6035 | 0.001 | 0.004** | -.24 |
| 2 | Cingulum Ant. | L | 24 | -3 | 33 | 10.5 | 3.56 | 4487 | 0.011 | 0.044* | -.18 |
| 3 | Cerebellum Crus I | R | 19 | 42 | -76.5 | -31.5 | 3.99 | 4266 | 0.005 | 0.020* | -.21 |
| 4 | Frontal Sup. | L | 32 | -21 | 13.5 | 42 | 5.08 | 1771 | 0.002 | 0.008** | -.26 |

The table shows the results from an analogous TFCE analysis as the one presented in the main text (**Table 1**) but excluding other psychopathology symptoms subscales as covariates, namely, excluding emotion, conduct and peer problems. As can be noted from **Table S2** and **Figure S4** (below), the significant brain clusters with and without these covariates are almost identical suggesting that the inclusion of the other symptom subscales does not appear to change the overall pattern of our findings. In addition, excluding the other subscales did not alter the failure to detect significant sex-by-psychopathology interactions for the other subscales: conduct, emotion and peer problems. Unadjusted *p*-values are corrected for the FWE within the brain statistical map at an alpha=0.05, adjusted *p*-values are additionally corrected for experiment-wise multiple comparisons across the four subscales using Bonferroni correction. The adjusted *p*-values that are statistically significant are marked with ** (p<0.01) and * (p<0.05). Anatomical descriptions were achieved using the AAL (Anatomical Automatic Labelling) and BA (Brodmann Area) atlases provided in MRIcron v.2016 ([www.nitrc.org/projects/mricron](http://www.nitrc.org/projects/mricron)) ([Rorden, Karnath, & Bonilha, 2007](#_ENREF_6)). k=cluster size; *df*=1510; *p*-value (2-tailed) with alpha=0.05.


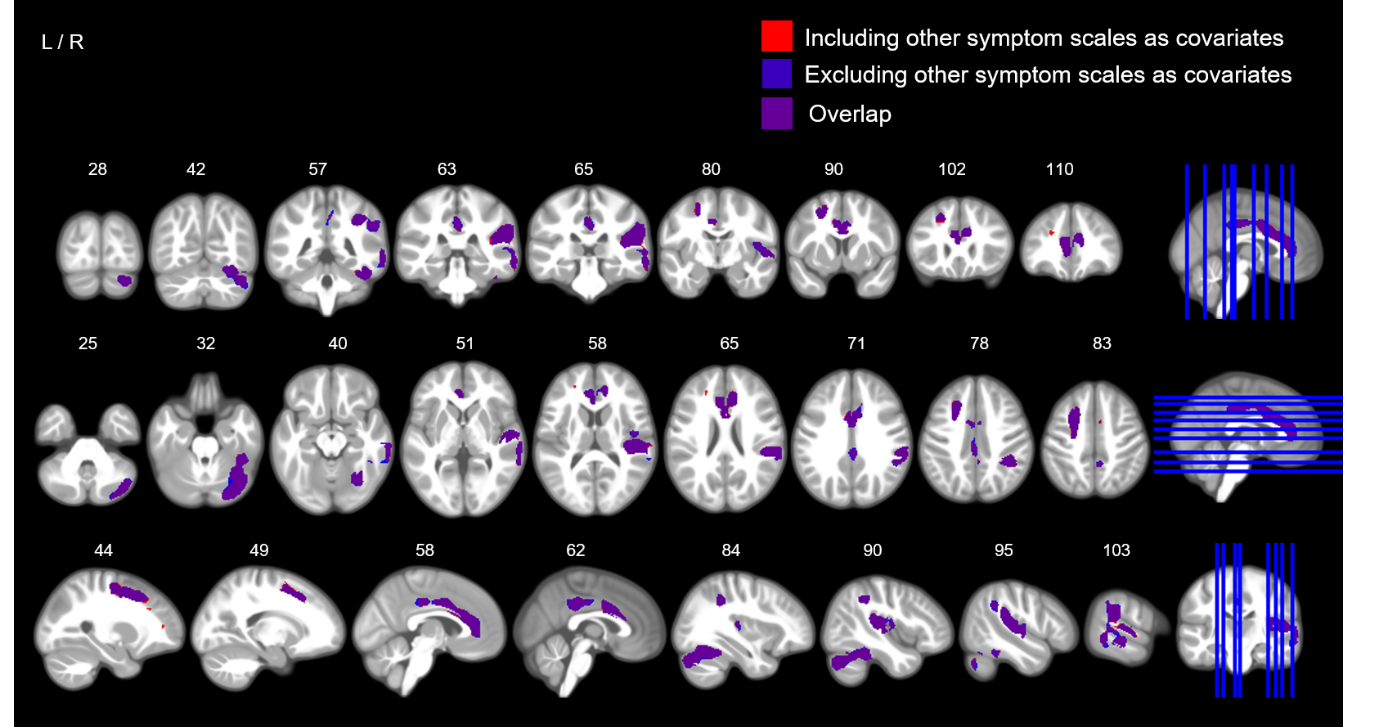


**Figure S4 Thresholded maps of significant brain regions for TFCE analyses including and excluding psychopathology symptom subscales as covariates.** The figure shows the sex-by-psychopathology interaction in GMV, including (red) and excluding (blue), the other psychopathology symptom subscales as covariates. The colour purple shows the overlap of these two maps, which is extensive and, suggests a minimal difference in the results between the two variants of the analysis. The red map is equivalent to the one shown in **Figure 3**.


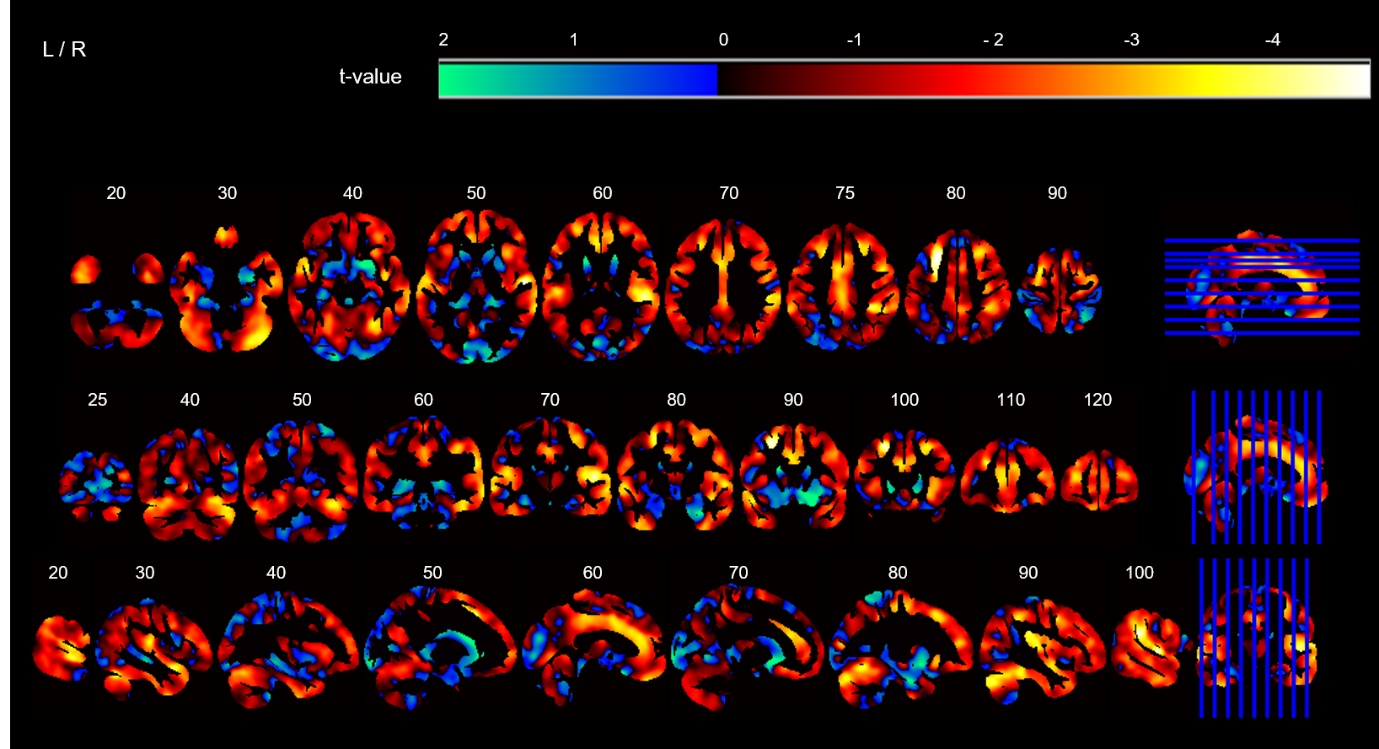


**Figure S5** **Unthresholded *t*-map corresponding to Figure 3.** The image illustrates coronal (top row), axial (middle row) and sagittal slices (bottom row) of the unthresholded *t*-statistic map at voxel-level for the main TFCE analysis which resulted in a significant sex-by-hyperactivity/inattention interaction effect. The cool colours (green-blue) indicate a positive association (positive *t*-statistics) and the warm colours (red-white) indicate a negative association (negative *t*-statistics) between GMV and sex-by-hyperactivity/inattention, whilst controlling for covariates (see **Figure S3** for design matrix).


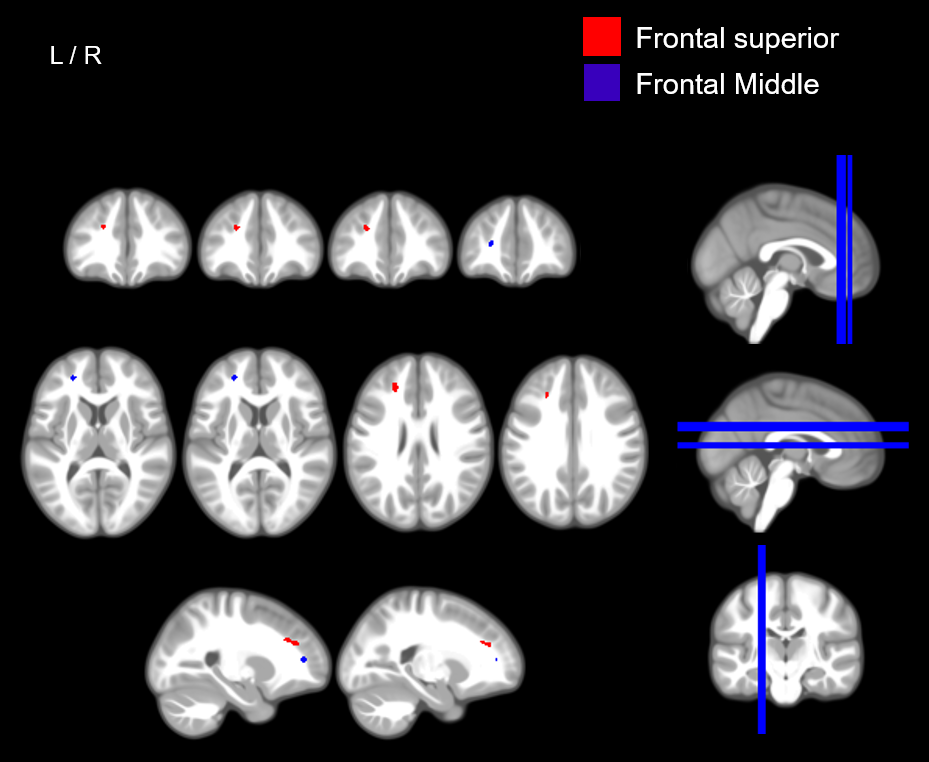


**Figure S6 Non-significant clusters for the sex-by-hyperactivity/inattention.** The image shows coronal (top row), axial (middle row) and sagittal slices (bottom row) of a thresholded *t*-map for the sex-by-hyperactivity/inattention interaction in GMV of the two clusters, Frontal superior and Frontal medial, which survived after correcting for the brain-wide FWE but did not survive the additional experiment-wise Bonferroni correction. See **Table 2** for details.

**Table S3a GLM results for sex differences in behavioural psychopathology symptom data (N=1526).**

|  | **Hyperactivity/inattention**  **problems** | **Conduct problems** | **Emotional difficulties** | **Peer Problems** |
| --- | --- | --- | --- | --- |
| Overall Regression model^ | *F*(15,1510) =23.3, *R*^2^ = .19,  *p* =2.56*10^-58^, *p_adj_* = 1.02*10^-57^ | *F*(15,1510) = 29.3, *R*^2^ =.23,  *p* =3.47*10^-73^, *p_adj_* = 1.39*10^-72^ | *F*(15,1510) = 31.7, *R*^2^ = .24,  *p* = 5.43*10^-79^, *p_adj_* = 2.17*10^-78^ | *F*(15,1510) =18.4, *R*^2^ = .15,  *p* = 1.86*10^-45^, *p_adj_* = 7.44*10^-45^ |
| Coefficient for sex variable | *β* = -.01, *r* =- .01, *d* = -0.02,*p* =.70 (n.s.) | *β* = .13, *r* = .12, *d* = 0.24,  *p* = 3.0*10^-6^, *p_adj_* = 1.2*10^-5^ | *β* = -.36, *r* = -.33, *d* = -0.69,  *p* = 1.54*10^-39^, *p_adj_* =6.15*10^-39^ | *β* = .17, *r* = .15, *d* = 0.30,  *p* = 5.54*10^-9^, *p_adj_* =2.21*10^-8^ |

**Table S3b GLM results for sex differences in behavioural psychopathology symptom data (N=2046)**

|  | **Hyperactivity/inattention**  **problems** | **Conduct problems** | **Emotional difficulties** | **Peer Problems** |
| --- | --- | --- | --- | --- |
| Overall Regression model^ | *F*(15,2030) =34.4, *R*^2^ = .20,  *p* =1.00*10^-88^, *p_adj_* = 4.01*10^-88^ | *F*(15,2030) = 40.3, *R*^2^ =.23,  *p* =2.00*10^-103^, *p_adj_* = 8.01*10^-103^ | *F*(15,2030) = 42.6, *R*^2^ = .24,  *p* = 5.23*10^-109^, *p_adj_* = 2.09*10^-108^ | *F*(15,2030) =23.7 ,*R*^2^ = .15,  *p* = 7.90*10^-61^, *p_adj_* = 3.16*10^-60^ |
| Coefficient for sex variable | *β* = -.02, *r* =- .02, *d* = -0.03,  *p* = .45(n.s.) | *β* = .15, *r* = .13, *d* = 0.27,  *p* = 1.44*10^-9^, *p_adj_* = 5.76*10^-9^ | *β* = -.36,*r* = .32, *d* = -0.69,  *p* = 2.25*10^-51^, *p_adj_* = 8.99*10^-51^ | *β* = .18,*r* = .11, *d* = 0.23,  *p* = 2.03*10^-7^, *p_adj_* = 8.12*10^-7^ |

**Table S3c GLM results for sex differences in behavioural psychopathology symptom data without subscales (N=1526)**

|  | **Hyperactivity/inattention**  **problems** | **Conduct problems** | **Emotional difficulties** | **Peer Problems** |
| --- | --- | --- | --- | --- |
| Overall Regression model^ | *F*(12,1513) =3.8, *R*^2^ = .03,  *p* =1.00*10^-5^, *p_adj_* = 8.00*10^-5^ | *F*(12,1513) = 5.3, *R*^2^ =.04,  *p* =8.71*10^-9^, *p_adj_* = 6.97*10^-8^ | *F*(12,1513) = 16.6, *R*^2^ = .11,  *p* = 9.85*10^-34^, *p_adj_* = 7.88*10^-33^ | *F*(12,1513) =5.5 ,*R*^2^ = .04,  *p* = 2.51*10^-9^, *p_adj_* = 2.01*10^-8^ |
| Coefficient for sex variable | *β* = .16, *r* =- .01, *d* = -0.01,  *p* =.84(n.s.) | *β* = .10, *r* = .08, *d* = 0.17,  *p* = 1.00*10^-3^, *p_adj_* = 8.00*10^-3^ | *β* = -.33,*r* =- .28, *d* = -0.58,  *p* = 4.67*10^-29^, *p_adj_* =3.73*10^-28^ | *β* = .10, *r* = .08, *d* = 0.17,  *p* = 5.62*10^-3^, *p_adj_* =.045 |

**Tables S3a-c** show different versions of the four GLM analyses that were run to test sex differences in the behavioural psychopathology symptom score data based on the SDQ questionnaire, one for each subscale. In each GLM, the dependent variable is the relevant psychopathology symptom score, the covariate of interest is sex and, the other covariates are the following: site, handedness, age, puberty, SES and the remaining three other psychopathology subscales (except for **Table S3c** where the latter psychopathology subscales covariates were excluded). For example, in **Table S3a and S3c,** the GLM’s dependent variable was hyperactivity/inattention problems, the covariate of interest was sex and the other covariates were the following: site, handedness, age, SES, conduct problems, emotional difficulties and peer problems. **Table S3a** shows the full model output as presented in the main paper with an N=1526. **Table S3b** shows the same GLM but run using the maximum number of complete cases available for the behavioural data (N=2046). **Table S3c** shows the same model as **Table S2a** (N=1526) but excluding the other psychopathology symptom subscales as covariates. Conduct problems, peer problems and emotional difficulties showed significant sex differences (measures of central tendency for the sample of N=1526 are available in **Figure 2**). Conduct problems (externalising) and peer problems (internalising) were higher in boys than girls, and emotional difficulties (internalising) were significantly higher in girls. No significant sex differences were detected for hyperactivity/inattention*.* These findings did not change (in terms of statistically significant sex differences) across the three versions presented (**Tables** **S3a-c**). *R*^2^= variance explained; *F*(df,df)= F-statistic (degrees of freedom regression, degrees of freedom residual); *β*=standardized beta coefficient; *r*=Pearson’s coefficient; *d*=Cohen’s d; n.s.= not statistically significant, *p_adj=_*Bonferroni-adjusted *p*-value.

# Part 5: Post-hoc tests

## Post-hoc test 2: global GMV

To test if any interaction effects were detectable at a global, not just voxel-level, a second post-hoc test used a GLM using whole-brain GMV, rather than voxel-level GMV, as the dependent variable and sex-by-hyperactivity/inattention as the covariate of interest and sex, site, handedness, age, puberty score, SES, TIV, hyperactivity/inattention, conduct, peer and emotion problems as additional covariates.

## Post-hoc test 3: covarying normalised brain volume

A third post-hoc test investigated whether the sex-by-hyperactivity/inattention GMV interaction within the significant clusters remained significant even after covarying out for an additional measure of brain volume, normalised brain volume. Normalised brain volume for each participant was calculated as the sum of grey matter and white matter, divided by TIV i.e. (GM+WM)/(GM+WM+CSF). The GLM used the GMV extracted from all significant brain clusters as the dependent variable, sex-by-hyperactivity/inattention as the covariate of interest and sex, site, handedness, age, puberty score, SES, TIV, normalised brain volume, hyperactivity/inattention, conduct, peer and emotion problems as covariates.

# References

Ashburner, J. (2007). A fast diffeomorphic image registration algorithm. *NeuroImage, 38*(1), 95-113. doi:<https://doi.org/10.1016/j.neuroimage.2007.07.007>

Ashburner, J., & Friston, K. J. (2005). Unified segmentation. *NeuroImage, 26*(3), 839-851. doi:<https://doi.org/10.1016/j.neuroimage.2005.02.018>

Goodman, R. (1997). The strengths and difficulties questionnaire: A research note. *Journal of Child Psychology and Psychiatry and Allied Disciplines, 38*(5), 581-586. doi:<https://doi.org/10.1111/j.1469-7610.1997.tb01545.x>

Goodman, R., Ford, T., Richards, H., Gatward, R., & Meltzer, H. (2000). The Development and Well-Being Assessment: Description and initial validation of an integrated assessement of child and adolescent psychopathology. *Journal of Child Psychology and Psychiatry and Allied Disciplines, 41*(5), 645-655. doi:<https://doi.org/10.1017/S0021963099005909>

Penny, W., Friston, K., Ashburner, J., Kiebel, S., & Nichols, T. (2007). *Statistical Parametric Mapping: The Analysis of Functional Brain Images*.

Rorden, C., Karnath, H. O., & Bonilha, L. (2007). Improving Lesion-Symptom Mapping. *Journal of Cognitive Neuroscience, 19*(7), 1081-1088. doi:<https://doi.org/10.1162/jocn.2007.19.7.1081>

WISC-IV. (2003). In: Fourth edition. San Antonio, Tex. : Psychological Corp., [2003] ©2003.
